# Breastfeeding cessation and symptoms of anxiety and depression: a longitudinal cohort study

**DOI:** 10.1186/1471-2393-12-36

**Published:** 2012-05-23

**Authors:** Eivind Ystrom

**Affiliations:** 1 Department of Adult Mental Health, Norwegian Institute of Public Health, P.O.box 4404, N-0403, Oslo, Norway

## Abstract

**Background:**

Neonatal anxiety and depression and breastfeeding cessation are significant public health problems. There is an association between maternal symptoms of anxiety and depression and early breastfeeding cessation. In earlier studies, the causality of this association was interpreted both ways; symptoms of anxiety and depression prepartum significantly impacts breastfeeding, and breastfeeding cessation significantly impacts symptoms of anxiety and depression.

First, we aimed to investigate whether breastfeeding cessation is related to an increase in symptoms of anxiety and depression from pregnancy to six months postpartum. Second, we also investigated whether the proposed symptom increase after breastfeeding cessation was disproportionately high for those women already suffering from high levels of anxiety and depression during pregnancy.

**Methods:**

To answer these objectives, we examined data from 42 225 women in the Norwegian Mother and Child Cohort Study (MoBa). Subjects were recruited in relation to a routine ultra-sound examination, and all pregnant women in Norway were eligible. We used data from the Medical Birth Registry of Norway and questionnaires both pre and post partum. Symptoms of anxiety and depression at six months postpartum were predicted in a linear regression analysis by WHO-categories of breastfeeding, symptoms of anxiety and depression prepartum (standardized score), and interaction terms between breastfeeding categories and prepartum symptoms of anxiety and depression. The results were adjusted for cesarean sections, primiparity, plural births, preterm births, and maternal smoking.

**Results:**

First, prepartum levels of anxiety and depression were related to breastfeeding cessation (*β* 0.24; *95% CI* 0.21-0.28), and breastfeeding cessation was predictive of an increase in postpartum anxiety and depression ( *β* 0.11; *95%CI* 0.09-0.14). Second, prepartum anxiety and depression interacted with the relation between breastfeeding cessation and postpartum anxiety and depression ( *β* 0.04; *95% CI* 0.01-0.06). The associations could not be accounted for by the adjusting variables.

**Conclusions:**

Breastfeeding cessation is a risk factor for increased anxiety and depression. Women with high levels of anxiety and depression during pregnancy who stop breastfeeding early are at an additional multiplicative risk for postpartum anxiety and depression.

## Background

Negative affectivity [1], refers to an individual’s disposition to states of fear, distress, sadness, panic, and fatigue. When exposed to stress, persons with high levels of negative affectivity are prone to experience symptoms of anxiety and depression [2]. While symptoms of anxiety comprise unpleasant and fearful states characterized by high arousal and activation, symptoms of depression comprise states characterized by feelings of sadness, hopelessness, fatigue, and anhedonia.[3] Persons experiencing symptoms of anxiety and depression are also more prone to experience states of irritability and anger.[1-3] Symptoms of depression lasting more than two weeks and relating to most aspects of daily life, called major depressive episode or often merely depression [3], is associated with severe outcomes for the individual, and are risk factors for all major disease-related causes of death [4]. Furthermore, postpartum anxiety and depression is a risk factor for developmental delays and internalizing problems in the child [5]. Mothers with high levels of anxiety and depression are more likely to give supplementary nutrition and less likely to sustain breastfeeding [6-13] throughout the recommended six months postpartum [14]. Sustained breastfeeding promotes sensory and cognitive development, and protects the infant against infectious and chronic diseases [15,16]. Furthermore, breastfeeding cessation is associated with an increased risk for type 2 diabetes mellitus, breast cancer, and ovarian cancer. [17] The general association between maternal negative affect and breastfeeding cessation has been interpreted in several ways. The first interpretation is that breastfeeding has anxiolytic and anti-depressive effects due to oxytocin [18]. This conclusion has been corroborated in controlled studies [19]. However, trait anxiety and depression measured prepartum has a substantial hampering impact on breastfeeding in the first six months postpartum; therefore, the association has also been interpreted as due to inefficient coping [14,20].

It is unclear whether breastfeeding cessation is predictive of anxiety and depression at six months postpartum beyond the initial anxiety and depression measured prepartum. A positive finding would indicate breastfeeding cessation is associated with an increase in anxiety and depression. Also, if breastfeeding cessation accounts for changes in symptoms of anxiety and depression, to what extent are mothers who are vulnerable to stress (i.e., having high initial levels of anxiety and depression) even more prone to an increase in symptoms of anxiety and depression due to breastfeeding cessation (i.e., an interaction effect). Individuals with high levels of anxiety and depression are more stress vulnerable [21,22]; therefore, such an interaction effect could indicate stress vulnerable mothers are not only more prone to stop breastfeeding, but also more prone to not cope well with breastfeeding cessation.

In the current study we aim to, first, investigate whether breastfeeding cessation is related to an increase in symptoms of anxiety and depression from pregnancy to six months postpartum. Second, we also investigated whether the proposed symptom increase after breastfeeding cessation was disproportionately high for those women already suffering from high levels of anxiety and depression during pregnancy.

## Methods

### Study population

The sample was taken from the Norwegian Mother and Child Cohort Study (MoBa), a prospective population-based pregnancy cohort study performed by the Norwegian Institute of Public Health [23]. Except two hospitals, all hospitals and maternity units in Norway with more than 100 births annually, altogether 50 units, were included [23]. There were no exclusion criteria for the women to be included into the study; all pregnant women were eligible. Expecting mothers were invited to join the study through postal invitation in connection with a routine ultrasound examination offered to all pregnant women in Norway at gestation weeks 17–18, and 42.7% agreed to participate. The assessment points were at 17 weeks gestation, 30 weeks gestation, and six months postpartum. At these time points the mothers were sent questionnaires containing questions on their physical health, mental health, nutritional status, and demographic status. For those invited to the study, the response rate during pregnancy ranged from 92% to 95%, and the response rate at six months postpartum was 87%. The MoBa is ongoing, and the MoBa-study group annually releases quality-assured data files. The current study was based on version three of the quality-assured data files released for research in 2007. Informed consent was obtained from each participant. The MoBa has been granted by the Norwegian Parliament [23] has a license from the Norwegian National Data Inspectorate (license 01/4325). Furthermore, the study was considered by the regional committee for ethics in medical research for South-eastern Norway, and received a positive ethical consideration (S-97045; S-95113). In addition to questionnaire data, we retrieved medical information on parturition for the present study from the Medical Birth Registry of Norway (MBRN). This registry contains information about all births in Norway [24].

### Sample

At the time of the present study, 47 659 mothers had returned the questionnaires at both 30 weeks gestation and six months postpartum. This number included only the last enrollment of a mother in the study and excluded previous participation with earlier pregnancies. Moreover, in the case of plural births, we included only one of the twins to avoid dependence between observations. Among the 47 659 mothers 5434 had missing data on one or more variables. Missing data among these mothers was distributed as follows: maternal age, 3; breast milk and solids, 3508; plural birth, 240; preterm birth, 33; daily smoking, 2045. The 5434 mothers with missing data were excluded from the analysis.

### Measures

The variables included in the data analysis were primiparity, plural births, cesarean sections, and gestational age (based on ultrasound examination) from the MBRN. When information was missing in the MBRN, we used self reported data from the questionnaire at six months postpartum. We collected information on symptoms of anxiety and depression, breastfeeding, introduction of solids, and daily smoking from the questionnaires. We defined daily smoking as smoking one or more cigarettes per day during the first six months postpartum. Preterm birth was defined as parturition before 37 weeks gestation.

We assessed maternal anxiety and depression symptoms at 30 weeks gestation and six months postpartum using a short version of the Hopkins Symptom Checklist (SCL-8) [25]. The SCL-8 is an 8-item self report instrument designed to assess psychological distress, in particular anxiety and depression. The response categories range from one to four (*not bothered* to *very much bothered*). The SCL-8 had internal consistencies of α = 0.84 at 30 weeks gestation and α = 0.86 at six months postpartum. We computed the average score across the eight items. For the multivariate analyses, we standardized the scores. Each interval then represents a shift of one standard deviation.

The introduction of and sustainment of breastfeeding, bottle feeding, and solids was reported by the mothers month by month at six months postpartum. Breastfeeding was categorized into three groups: predominant breastfeeding, mixed breastfeeding, and bottle-feeding. This is largely in accordance with the classification system of *the World Health Organization*[26]. This categorization is described in detail in an earlier study on breastfeeding derived from this cohort [14]. Predominant breastfeeding is when the infant’s predominant source of nutrition is breast milk. Mixed breastfeeding is continued breastfeeding up to six months postpartum, supplemented by formula or solids. Bottle-feeding referred to those mothers who stopped breastfeeding completely and used only milk supplementation and solids.

### Statistics

We imputed missing data on the SCL-8 by applying the estimation-maximization algorithm [27]. The imputed data was used across all analyses.

To investigate main effects and interaction effects, we used linear regression with several blocks, always keeping variables from previous blocks. Mixed breastfeeding and bottle-feeding comprise together the alternatives to full breastfeeding. Therefore they were added together in each block. The first block was the main effects of mixed breastfeeding and bottle-feeding on symptoms of anxiety and depression at six months postpartum. In the second block we investigated the effect of mixed breastfeeding and bottle-feeding on the change in symptoms of anxiety and depression by introducing the anxiety and depression symptom score assessed at 30 weeks gestation. In the third block, we investigated any additional interactive effects over and beyond the additive effects, and introduced the interaction term between mixed breastfeeding and symptoms of anxiety and depression at 30 weeks gestation and the interaction term between bottle-feeding and symptoms of anxiety and depression at 30 weeks gestation. In the forth block, we introduced adjusting variables related to events happening from parturition to six months postpartum (i.e. cesarean sections, primiparity, plural births, preterm births, and daily smoking). All adjusting variables were entered in a single block to investigate if they as a whole could account for the associations in the previous blocks. The blocks were expanded by the following theoretical rationale: First block, to establish that breastfeeding cessation is associated with post partum symptoms of anxiety and depression; second block, to be the cause of increase in symptoms of anxiety and depression, breastfeeding cessation must be associated with post partum symptoms of anxiety and depression after adjusting for prepartum symptom level; third block, if women with high prepartum levels of anxiety and depression are more vulnerable to the detrimental effects of breastfeeding cessation, there must be an interaction effect between prepartum symptom level and breastfeeding cessation; fourth block, if the interaction effect could merely be a consequence of other contextual factors known to influence breastfeeding, therefore a handful of these factors were adjusted for.

To limit multicollinearity, increase normality, and enhance interpretability, the SCL-8 scores were centered, log-transformed, and standardized. We used an alpha of 0.05 across the analyses.

## Results

Among the mothers, 13.9% had undergone a cesarean section, 10.7% were smoking while pregnant, 1.9% had a plural delivery, 5.5% came into labor preterm, and 42.2% were primiparas (Table 1). The following percentages of the 42 225 women were fully breastfeeding, mixed breastfeeding, and bottle feeding the first five months postpartum: 1^st^ month: 83.2%, 15.5%, 1.4%; 2^nd^ month: 77.8%, 18.8%, 3.3%; 3^rd^ month: 71.8%, 22.5%, 5.8%; 4^th^ month: 63.1%, 27.9% 9.0%; and 5^th^ month: 39.1%, 48.1%, 12.8% (Table 1). At six months postpartum, 15.1% of the mothers were still fully breastfeeding, 68.8% were giving solid food or formula in addition to breastfeeding, and 16.1% had stopped breastfeeding entirely (Table 1). The average (±*SE*) SCL-8 score in mothers who conducted predominant breastfeeding, mixed breastfeeding, and bottle feeding was 1.24±0.004, 1.26±0.002, and 1.32±0.005 prepartum, and 1.22±0.004, 1.24±0.002, and 1.31±0.005 postpartum.

**Table 1:** Characteristics of the 42 225 mothers included in the analyses*

Age (y)	30.1 ± 4.5 †
Anxiety/depressiveness 30th week of gestation§	1.27 ± 0.34
Anxiety/depressiveness 6 months postpartum§	1.25 ± 0.35
Bottle feeding (%)	
1^st^ month post partum	581 (1.4) [162]
2^nd^ month post partum	1405 (3.3) [114]
3^rd^ month post partum	2438 (5.8) [98]
4^th^ month post partum	3797 (9.0) [92]
5^th^ month post partum	5380 (12.8) [74]
6^th^ month post partum	6803 (15.1)
Cesarean section (%)	5857 (13.9)
Maternal smoking (%)	4532 (10.7)
Mixed breastfeeding (%)	
1^st^ month post partum	6503 (15.5) [162]
2^nd^ month post partum	7924 (18.8) [114]
3^rd^ month post partum	9461 (22.5) [98]
4^th^ month post partum	11 736 (27.9) [92]
5^th^ month post partum	20 276 (48.1) [74]
6^th^ month post partum	29 057 (68.8)
Plural Birth (%)	823 (1.9)
Predominant breastfeeding (%)	
1^st^ month post partum	34 979 (83.2) [162]
2^nd^ month post partum	32 782 (77.8) [114]
3^rd^ month post partum	30 228 (71.8) [98]
4^th^ month post partum	26 600 (63.1) [92]
5^th^ month post partum	16 495 (39.1) [74]
6^th^ month post partum	6365 (16.1)
Preterm birth (%)	2320 (5.5)
Primiparous (%)	17 837 (42.2)

* Number of cases in brackets.

† Mean ± SD (all such values).

‡ Number of cases with missing data at that time point in square brackets (all such values).

In the first block of the linear regression analysis, standardized symptoms of anxiety and depression at six months postpartum were predicted by mixed breastfeeding (*β* 0.08; *95% CI* 0.05-0.11) and bottle feeding ( *β* 0.24; *95% CI* 0.21-0.28) at the same time point (Table 2). The non-overlapping confidence intervals means that bottle feeding is a more severe risk factor for anxiety and depression than mixed breastfeeding.

**Table 2:** Linear regression analysis where anxiety/depressiveness 6 months post partum is predicted by infant feeding (block 1); infant feeding and baseline anxiety/depressiveness (block 2); infant feeding, baseline anxiety/depressiveness, and interaction terms (block 3); and infant feeding, baseline anxiety/depressiveness, interaction terms, and medical adjusting variables (block 4)*

**Block**		***Adj β***	***(95% CI)***	***R***^*2*^	***p R***^*2*^***change***
1	Intercept (predominant breastfeeding)	−0.10	(−0.12 - -0.07)	.005	.000
	Mixed breastfeeding	0.08	(0.05 - 0.11)		
	Bottle feeding	0.24	(0.21 - 0.28)		
2	Intercept (predominant breastfeeding)	−0.05	(−0.07 - -0.03)	.315	.000
	Mixed breastfeeding	0.04	(0.02 - 0.06)		
	Bottle feeding	0.11	(0.09 - 0.14)		
	Anxiety/depressiveness 30^th^ week of gestation†	0.56	(0.55 - 0.57)		
3	Intercept (predominant breastfeeding)	−0.05	(−0.07 - -0.03)	.316	.002
	Mixed breastfeeding	0.04	(0.02 - 0.07)		
	Bottle feeding	0.11	(0.08 - 0.14)		
	Anxiety/depressiveness 30^th^ week of gestation†	0.55	(0.53 - 0.57)		
	Anxiety/depressiveness 30^th^ week of gestation x mixed breastfeeding (interaction term)	0.00	(−0.02 - 0.02)		
	Anxiety/depressiveness 30^th^ week of gestation x bottle feeding (interaction term)	0.04	(0.01 - 0.06)		
4	Intercept (predominant breastfeeding)	−0.03	(−0.06 - -0.01)	.317	.000
	Mixed breastfeeding	0.04	(0.02 - 0.07)		
	Bottle feeding	0.10	(0.07 - 0.13)		
	Anxiety/depressiveness 30^th^ week of gestation†	0.55	(0.53 - 0.57)		
	Anxiety/depressiveness 30^th^ week of gestation x mixed breastfeeding (interaction term)	0.00	(−0.02 - 0.02)		
	Anxiety/depressiveness 30^th^ week of gestation x bottle feeding (interaction term)	0.03	(0.01 - 0.06)		
	Cesarean section	−0.01	(−0.04 - 0.01)		
	Primiparous	−0.05	(−0.06 - -0.03)		
	Plural Birth	0.03	(−0.03 - 0.09)		
	Preterm birth	0.01	(−0.03 - 0.04)		
	Maternal smoking	0.08	(0.05 - 0.10)		

* Standardized log-transformed score of SCL-8 (i.e. anxiety/depressiveness) 6 months post partum as dependent variable.

† Standardized log-transformed score of SCL-8.

In the second block, we tested whether mixed breastfeeding and bottle-feeding were associated with greater postpartum symptoms of anxiety and depression than found prepartum. When we added the baseline score in this second block, the effect of mixed breastfeeding and bottle-feeding was reduced to (*β* 0.04; *95% CI* 0.02-0.06) and ( *β* 0.11; *95%CI* 0.09-0.14), respectively (Table 2). First, this means that adjusting for initial level of anxiety and depression reduced the difference between the child feeding groups to about half. Second, it means that the remaining difference in anxiety and depression could be attributed to change from 30^th^ week of gestation to six months post partum. Third, the non-overlapping confidence intervals means that bottle feeding is a more severe risk factor than mixed breastfeeding for change in anxiety and depression the first six months post partum.

In the third block, we added the two interaction terms between baseline symptoms of anxiety and depression and the two forms of infant feeding. There was a positive interaction effect between bottle-feeding and baseline symptoms of anxiety and depression on changes in symptoms of anxiety and depression (i.e. change from baseline to six months postpartum) (Table 2). This means that the change in anxiety and depression for women who bottle feeds increases with 0.04 for each SD of prepartum anxiety and depression. The interaction effect was not significant for mixed breastfeeding.

In the fourth block, we added medical adjusting variables related to events during the postpartum period to investigate if those other events were the underlying causes of breastfeeding cessation. While cesarean sections, plural births, and preterm births were not related to changes in anxiety and depression, primiparity was related to a decrease in symptoms of anxiety and depression and maternal smoking to an increase in symptoms of anxiety and depression (Table 2). None of the variables from the three earlier blocks, including the interaction terms, were made redundant due to the medical adjusting variables.

## Discussion

Our first finding in this study was that while 15.1% of the mothers were still fully breastfeeding six months postpartum, 68.8% were giving solid food or formula in addition to breastfeeding, and 16.1% had stopped breastfeeding entirely. Second, mixed breastfeeding and bottle-feeding were related to higher levels of anxiety and depression at six months postpartum. This corresponds to findings from earlier studies [6-10][11-13] and earlier findings using the MoBa cohort [14]. However, this association has also been inconsistent in other studies [10,28].

We also found that after adjusting for baseline prepartum anxiety and depression, the effect of infant feeding on postpartum anxiety was reduced by half, but was still significant. This suggests the conclusions that breastfeeding is related to a reduction in anxiety and depression [10,19] in mothers and prepartum anxiety and depression are related to breastfeeding cessation [10,14] are both valid. Furthermore, we tested if a high initial level of anxiety and depression at baseline would accelerate the detrimental effect of mixed breastfeeding or breastfeeding cessation. Only bottle-feeding (i.e. breastfeeding cessation) interacted with symptoms of anxiety and depression at baseline on changes in symptoms of anxiety and depression. These findings can be explained as follows: Imagine two women, one with an average level of anxiety and depression prepartum and one with a high level of prepartum anxiety and depression. Imagine further that they both stop breastfeeding. The results from block two implicate that both women would have higher anxiety and depression at six months postpartum than they had prepartum. The implication from the finding on the interaction term (block 3) is that the second woman (high baseline level of anxiety and depression) would have an even larger increase in anxiety and depression as the first woman (average baseline level of anxiety and depression). This could be because individuals with high levels of anxiety and depression are more vulnerable to stress and are more prone to depressive episodes [22]. The findings from the three earlier blocks were not made redundant by the medical adjusting variables of cesarean section, primiparity, plural birth, preterm birth, and maternal smoking.

An important limitation to consider when generalizing our findings is that other unmeasured events could be the underlying cause of breastfeeding cessation and, therefore, the real predictor of changes in symptoms of anxiety and depression. In a recent study it was found that women with severe breastfeeding discomfort were more likely to be depressed. [11] Breastfeeding pain could therefore be an example of a third variable leading to both depressive symptoms and breastfeeding cessation. Furthermore, it was found in the aforementioned study that breastfeeding protected against depression when subjected to pain. [11] In the current study we also found that breastfeeding gave protection against a risk factor for post partum depression.

## Conclusions

We found prenatal symptoms of anxiety and depression are linked to breastfeeding cessation to the same extent that breastfeeding cessation is linked to an increase in symptoms of anxiety and depression six months postpartum. In addition, women who have high levels of prenatal anxiety and depression are more prone than others to an increase in postnatal anxiety and depression after breastfeeding cessation. The main and interactive effects combined would for these women be of substantial clinical significance. The clinical implications of our study are that mothers characterized by anxiety and depression may need breastfeeding support and to learn efficient coping strategies in cases of unintended breastfeeding cessation. These findings are of importance to health professionals involved in the primary care of childbearing women. Future studies should investigate a full range of personality factors and coping strategies as predictors and moderators of breastfeeding behavior in an epidemiological sample.

## Competing interests

The author declares that he has no competing interests.

## Authors' contributions

EY carried out the statistical analyses, and drafted the manuscript. The author read and approved the final manuscript.

## Pre-publication history

The pre-publication history for this paper can be accessed here:

http://www.biomedcentral.com/1471-2393/12/36/prepub

## References

[B1] Watson D Clark LA Negative affectivity: the disposition to experience aversive emotional states Psychol Bull 1984 96 465 490 6393179

[B2] Clark LA Watson D John OP, Robins RW, Pervin LA Temperament Handbook of Personality 2008 The Guildford Press, New York 265 286

[B3] American Psychiatric Association Diagnostic and statistical manual of mental disorders 2000 4 Author, Washington, DC text rev. edn

[B4] Mykletun A Bjerkeset O Dewey M Prince M Overland S Stewart R Anxiety, Depression, and Cause-Specific Mortality: The HUNT Study Psychosom Med 2007 69 323 331 10.1097/PSY.0b013e31803cb862 17470669

[B5] Brand SR Brennan PA Impact of antenatal and postpartum maternal mental illness: How are the children? Clin Obstet Gynecol 2009 52 441 455 10.1097/GRF.0b013e3181b52930 19661760

[B6] Dunn S Davies B McCleary L Edwards N Gaboury I The relationship between vulnerability factors and breastfeeding outcome Jognn J Obstet Gynecol Neonatal Nurs 2006 35 87 97 10.1111/j.1552-6909.2006.00005.x 16466356

[B7] Galler JR Harrison RH Biggs MA Ramsey F Forde V Maternal moods predict breastfeeding in Barbados J Dev Behav Pediatr 1999 20 80 87 10.1097/00004703-199904000-00002 10219685

[B8] Galler JR Harrison RH Ramsey F Chawla S Taylor J Postpartum feeding attitudes, maternal depression, and breastfeeding in Barbados Infant Behav Dev 2006 29 189 203 10.1016/j.infbeh.2005.10.005 17138274

[B9] Henderson JJ Evans SF Straton JAY Priest SR Hagan R Impact of postnatal depression on breastfeeding duration Birth 2003 30 175 180 10.1046/j.1523-536X.2003.00242.x 12911800

[B10] Dennis CL McQueen K The relationship between infant-feeding outcomes and postpartum depression: a qualitative systematic review Pediatrics 2009 123 e736 10.1542/peds.2008-1629 19336362

[B11] Watkins S Meltzer-Brody S Zolnoun D Stuebe A Early breastfeeding experiences and postpartum depression Obstet Gynecol 2011 118 214 221 10.1097/AOG.0b013e3182260a2d 21734617

[B12] Thome M Alder EM Ramel A A population-based study of exclusive breastfeeding in Icelandic women: is there a relationship with depressive symptoms and parenting stress? Int J Nurs Stud 2006 43 11 20 10.1016/j.ijnurstu.2004.10.009 16326160

[B13] Papinczak TA Turner CT An analysis of personal and social factors influencing initiation and duration of breastfeeding in a large Queensland maternity hospital Breastfeeding Rev Prof Publ Nurs Mothers' Assoc Aust 2000 8 25 33 10842578

[B14] Ystrom E Niegel S Klepp K-I Vollrath ME The impact of maternal negative affectivity and general self-efficacy on breast-feeding: The Norwegian Mother and Child Cohort Study J Pediatr 2008 152 68 72 10.1016/j.jpeds.2007.06.005 18154903

[B15] Paricio Talayero JM Lizan-Garcia M Otero PA Benlloch Muncharaz MJ Beseler SB Sanchez-Palomares M Santos SL Rivera LL Full breastfeeding and hospitalization as a result of infections in the first year of life Pediatrics 2006 118 e92 e99 10.1542/peds.2005-1629 16818542

[B16] Kramer MS Aboud F Mironova E Vanilovich I Platt RW Matush L Igumnov S Fombonne E Bogdanovich N Ducruet T Collet JP Chalmers B Hodnett E Davidovslzy S Skugarevsky O Trofimovich O Kozlova L Shapiro S Breastfeeding and child cognitive development - New evidence from a large randomized trial Arch Gen Psychiatry 2008 65 578 584 10.1001/archpsyc.65.5.578 18458209

[B17] Ip S Chung M Raman G Trikalinos TA Lau J A Summary of the Agency for Healthcare Research and Quality's evidence report on breastfeeding in developed countries Breastfeeding Med 2009 4 S17 S30 10.1089/bfm.2008.0133 19827919

[B18] Uvnäs-Moberg K Widström AM Nissen E Björvell H Personality traits in women 4 days postpartum and their correlation with plasma levels of oxytocin and prolactin J Psychosom Obstet Gynecol 1990 11 261 273 10.3109/01674829009084422

[B19] Mezzacappa ES Katkin ES Breast-feeding is associated with reduced perceived stress and negative mood in mothers Health Psychol 2002 21 187 193 11950109

[B20] Ystrom E Maternal negative affectivity and child-feeding: Results from the Norwegian Mother and Child Cohort Study 2009 Unipub, Oslo

[B21] Vollrath M Personality and stress Scand J Psychol 2001 42 335 347 10.1111/1467-9450.00245 11547909

[B22] Kendler KS Kuhn J Prescott CA The interrelationship of neuroticism, sex, and stressful life events in the prediction of episodes of major depression Am J Psychiatry 2004 161 631 636 10.1176/appi.ajp.161.4.631 15056508

[B23] Magnus P Irgens LM Haug K Nystad W Stoltenberg C The MoBa Study Group The Norwegian Mother and Child Cohort Study Int J Epidemiol 2006 35 1146 1150 10.1093/ije/dyl170 16926217

[B24] Irgens LM The Medical Birth Registry of Norway. Epidemiological research and surveillance throughout 30 years Acta Obstet Gynecol Scand 2000 79 435 439 10.1080/j.1600-0412.2000.079006435.x 10857866

[B25] Strand BH Dalgard OS Tambs K Rognerud M Measuring the mental health status of the Norwegian population: A comparison of the instruments SCL-25, SCL-10, SCL-5 and MHI-5 (SF-36) Nord J Psychiatry 2003 57 113 118 10.1080/08039480310000932 12745773

[B26] World Health Organization Indicators for assessing breastfeeding practices, WHO/CDD/SER/91 1991 14 World Health Organization, Geneva

[B27] Schafer JL Graham JW Missing data: our view of the state of the art Psychol Meth 2002 7 147 177 12090408

[B28] Chaudron LH Klein MH Remington P Palta M Allen C Essex MJ Predictors, prodromes and incidence of postpartum depression J Psychosom Obstet Gynecol 2001 22 103 112 10.3109/01674820109049960 11446151

